# Expansion and Evolution of the X-Linked Testis Specific Multigene Families in the *melanogaster* Species Subgroup

**DOI:** 10.1371/journal.pone.0037738

**Published:** 2012-05-23

**Authors:** Galina L. Kogan, Lev A. Usakin, Sergei S. Ryazansky, Vladimir A. Gvozdev

**Affiliations:** Department of Molecular Genetics of Cell, Institute of Molecular Genetics, Russian Academy of Sciences, Moscow, Russia; University of Maryland School of Medicine, United States of America

## Abstract

The testis specific X-linked genes whose evolution is traced here in the *melanogaster* species subgroup are thought to undergo fast rate of diversification. The *CK2ßtes* and *NACβtes* gene families encode the diverged regulatory β-subunits of protein kinase CK2 and the homologs of β-subunit of nascent peptide associated complex, respectively. We annotated the *CK2βtes-like* genes related to *CK2ßtes* family in the *D. simulans* and *D. sechellia* genomes. The ancestor *CK2βtes-like* genes preserved in *D. simulans* and *D. sechellia* are considered to be intermediates in the emergence of the *D. melanogaster* specific *Stellate* genes related to the *CK2ßtes* family. The *CK2ßtes-like* genes are more similar to the unique autosomal *CK2ßtes* gene than to *Stellates*, taking into account their peculiarities of polymorphism. The formation of a variant the *CK2ßtes* gene *Stellate* in *D. melanogaster* as a result of illegitimate recombination between a *NACßtes* promoter and a distinct polymorphic variant of *CK2ßtes*-like ancestor copy was traced. We found a close nonrandom proximity between the dispersed defective copies of *DINE-1* transposons, the members of *Helitron* family, and the *CK2βtes* and *NACβtes* genes, suggesting an involvement of *DINE-1* elements in duplication and amplification of these genes.

## Introduction

The availability of genome sequences of related species permits to retrace the origination of new gene families [1]. New X-linked testis specific genes are thought to evolve frequently [2]–[4]. Recently, a role of the highly abundant transposable element *DINE-1* (also named *INE-1* and *DNAREP*1) in the emergence of these genes in the *Drosophila* genomes has been suggested [5]–[7]. Using available data sets of genome sequences from FlyBase [8], we traced the origination and amplification in the *melanogaster* subgroup species of the X-linked testes specific genes related to two multigene families, *CK2βtes* and *NACβtes*, encoding regulatory β-subunit of protein kinase CK2 and β-subunit of protein nascent associated complex (NAC), respectively. CK2 is a serine/threonine kinase that participates in a wide variety of cellular processes including cell differentiation, proliferation and survival [9]–[11]. The regulatory β-subunit ensures stability and specificity of CK2, and may also have functions distinct from CK2 as a component of some other protein kinases [9], [11]. Both conservative α- and β-subunits of NAC are known to contact with nascent polypeptide chains on the ribosome and contribute to the prevention of inappropriate interactions during the folding of nascent polypeptide [12]. The importance of *NACβ in vivo* function is emphasized by the early embryonically lethal *bicaudal* phenotype of a *NACß* mutant in *D. melanogaster* [13]. The testis specific functions of both CK2βtes and NACβtes proteins remain elusive.

*D. melanogaster* contains several paralogous CK2 protein kinase genes supposed to be involved in specification of CK2 targeting in cells [14]. The single autosomal gene on chromosome 2 encodes protein kinase CK2 regulatory β-subunit. The homologous amplified copies of the X-linked *Stellate* genes are normally silenced but have been shown to be expressed in the testes of *D. melanogaster* due to the absence of their Y-linked specific suppressors [14], [15]. The unique autosomal *CK2βtes* genes are located in homologous regions in the *D. melanogaster*, *D. sechellia*, *D. yakuba*, and *D. erecta* genomes according to FlyBase [8], while its presence in *D. simulans* requires a much more detailed analysis of convincing sequencing results. The amplified *Stellate* genes are found only in *D. melanogaster*, their derepression in testes leads to male sterility or semi-sterility owing to the abnormality of chromosome condensation and nondisjunction of sex chromosomes [16], [17]. Interest in *Stellate* genes has been inspired by the discovery of a RNA silencing mechanism of their repression [18]. The evolutionary significance of *Stellate* genes emergence remains an enigma, possibly their putative function is not limited to the modulation of protein kinase CK2 activity, but is also related to chromatin assembly [19]. Actually, protein kinase CK2 is predominantly a nuclear protein [9], Stellate protein has been detected in both cytoplasm and nucleus, and an ability of lysine methylated Stellate to mimic epitope of H3K9me3 histone has been shown [19]. This observation suggests a capacity of Stellate protein to compete with some chromatin “readers” of histone H3K9me3 mark. The emergence of the *CK2ßtes* family of *Stellate* gene has been driven by an acquisition of promoter from the *NACβtes* gene [20].

Here we annotated in *D. sechellia* and *D. simulans* several paralogous genes related to *CK2ßtes* family and designated as a new multigene family of *CK2ßtes*-*like* genes. The estimation of a similarity of these genes to the unique autosomal *CK2βtes* genes and *Stellate* genes in *D. melanogaster* allowed us to consider a putative *CK2ßtes*-*like* ancestor as an intermediate in the origination of *Stellate* genes. Although only single copy of the *NACβtes* gene is revealed in *D. yakub*a, similar patterns of the X-linked amplifications of *NACβtes* genes are detected in *D. melanogaster* and sister *D. simulans/D. sechellia* species. The copies of amplified *NACβtes* and *CK2βtes* gene families are localized in a restricted syntenic region (∼300–400 kb) in *D. melanogaster* and *D. simulans/D. sechellia*.

Using available genomic data sets of FlyBase [8] we demonstrated the juxtaposition of the repeated young X-linked *Stellate*, *CK2βtes-like* and *NACβtes* genes to polymorphic fragments of *DINE-1* transposable elements related to an enigmatic *Helitron* type. A close nonrandom location of *DINE-1*s to these amplified copies hints for *DINE-1* participation in the expansion of these protein-coding genes.

## Results and Discussion

**Figure 1:**
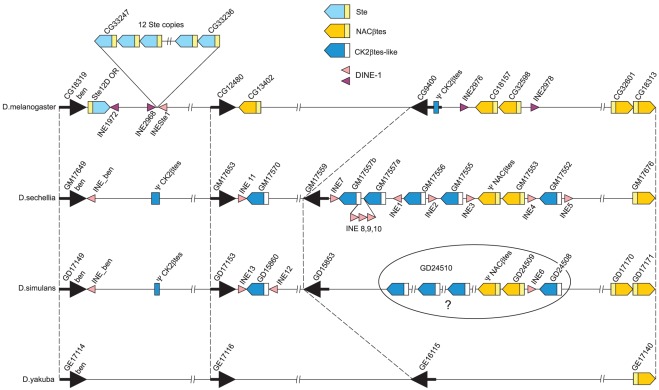
Scheme of syntenic X-chromosome regions comprising the *CK2βtes* and *NACβtes* multigene families in *Drosophila* species. The synteny is demonstrated by vertical dashed lines indicating positions of orthologous genes. The sizes of regions are ∼400 kb in *D. melanogaster* (X:13890387..14275449), ∼280–350 kb in *D. simulans* (X:10696104..10968610)*/D. sechellia* (scaffold_20:533142..877095) and ∼330 kb in *D.yakuba* (X:8186055..8516953). Positions of genes related to gene families are depicted by pentagons indicating direction of transcription. Yellow pentagons designate *NACβtes* copies, blue pentagons - *CK2ßtes*-*like* copies, light blue pentagons – *Stellate* genes. Promoters are indicated by small rectangles fused to these signs: light yellow rectangles depict homologous *Stellate* and *NACβtes* promoters, blue rectangles depict *CK2βtes-like* ones. Blue rectangles designate the remnants of *CK2βtes-like* sequences (*D. melanogaster* X:14189495..14189605 [-], *D. sechellia* scaffold_20: 574563..574 704[-], *D. simulans* X:10724910..10724990[-]). A remnant of *CK2βtes*-*like* gene represented by the ORF for 37 amino acids is designated in intron of gene CG9400 in *D. melanogaster*. Lilac and rose arrowheads designated earlier annotated and newly detected *DINE-1* elements, respectively. Orientations of arrowheads correspond to predicted direction of transcription. Positions of some orthologous genes are depicted by black arrows. In *D. simulans* several *CK2βtes*-*like* copies (GD24508:chrX_Mrandom_708:8043..8830[-], GD24510: chrX_Mrandom_706:885-1556[-]), *NACβtes* (GD24509:chrX_Mrandom_708:6003..6761[-]) and *ψNACβtes* gene are not attributed precisely to the studied syntenic region, these copies are enclosed in an oval frame.

The structures of syntenic regions of the X-chromosomes *of D. melanogaster*, closely related *D. sechellia/D. simulans* and *D. yakuba* are presented in Fig. 1. These regions contain *Stellate*, *CK2βtes-like* and *NACβtes* genes. The synteny is clearly demonstrated by relative positions of gene *bendless* (*ben*) as well as CG12480/GM17653/GD17153/GE17116 and CG9400/GM17559/GD15853/GE16115. The annotation procedure allowed us to present orthologs CG18313/GM17676/GD17171/GE17140 at the right border of the studied syntenic region. Paralogs CG18313/CG32601/CG32598/CG18157/CG13402 have been annotated earlier in *D. melanogaster* as *NACβtes* genes [20]. We have identified in the syntenic regions of the X-chromosomes in *D. simulans* and *D. sechellia* the *CK2βtes-like* genes related to autosomal *CK2βtes* gene (CG13591) in *D. melanogaster*. We found the fragments of *CK2βtes* genes (ψ*CK2βtes*) in *D. simulans* and *D. sechellia* at the same site where a cluster of *Stellate* genes is known to be emerged in *D. melanogaster*. The fragments of *DINE-1* elements were localized in syntenic region *of D. melanogaster*, *D. simulans* and *D. sechellia*.

The presented evolutionary tree of the representatives of the *CK2βtes* family. We traced the uprising of gene *Stellate* as a result of illegitimate recombination between the *NACβte*s promoter and a definite polymorphic variant of *CK2βtes-like* ancestor. At last we showed nonrandom associations of the remnants of *DINE-1* elements with *CK2βtes-like*, *Stellate* and *NACβte*s genes.

### The family of the *NACβtes* genes

The *NACßtes* genes in *D. melanogaster* (CG13402, CG18157, CG32598, CG32601 and CG18313) are indicated according to our earlier published data [20]. *D. melanogaster*, *D. sechellia* and *D. simulans* have several copies of highly homologous *NACßtes* genes but the *D. yakuba* genome contains only a single copy (GE17140). *D. simulans* and *D. sechellia* contain a pair of duplicated *NACβtes* copies similar to those in *D. melanogaster*, demonstrating their evolving in the common ancestor of these species. The *NACβtes* genes may be considered the young ones, due to their presence in the *melanogaster* subgroup species [20], but not in the *D. pseudoobscura* taking into account available data sets of FlyBase. The *NACßtes* pseudogenes are located adjacent to GM17553 and GD24509 in *D. sechellia* and *D. simulans*, respectively, but a complete sequence of *D. simulans* pseudogene is not yet available (Fig. 1, Fig. S1). The duplicated copies of *NACβtes* in *D. sechellia* are located in the same region in *D. melanogaster*, but in *D. sechellia* these genes are flanked by *CK2ßtes-like* copies (pair of genes GM17555/GM17556 and gene GM17552) (Fig. 1), forming a cluster of *NACßtes* and *CK2ßtes*-*like* genes.

### The family of the *CK2βtes* genes

The *CK2ßtes*-*like* copies comprise a new gene family represented by the variants of *CK2βtes* family genes that has been amplified in the *D. sechellia/D. simulans* lineage. The *CK2ßtes*-*like* genes are homologous to the unique autosomal *CK2ßtes* gene located in syntenic regions of the *D. melanogaster*, *D. sechellia* and *D. yakuba* genomes. The precise genomic structure of homologous region in *D. simulans* is not yet solved and only a single copy of *CK2ßtes-like* (GD24508) is annotated here. However, some unannotated *CK2ßtes*-*like* copies in *D. simulans* may be also attributed to this region (Fig. 1). The testis specific transcription of a representative of this family, GD24508 in *D. simulans*, was shown (Fig. S2). This observation allows us to consider this gene family as a testis specific one. *D. yakuba* contains no *CK2βtes*-*like* genes on the X-chromosome and elsewhere in the genome.

Multiple alignment of amino acid residues of proteins and phylogenetic tree related to CK2βtes family genes (CK2βtes, CK2βtes-like and Stellate) is shown in Fig. S3. The peculiarities of amino acid substitution patterns (Fig. S3A) as well as protein phylogenetic analysis (Fig. S3B) allow us to discriminate CK2βtes-like proteins as a distinct novel subfamily, and the phylogenetic tree demonstrates the origination of *Stellate* genes from *CK2βtes-like* ancestor.

The CK2 β-subunit is remarkably conserved among species [21], [22]. All CK2βtes subunits carry at their N-termini the site S2 of autoposphorylation known to be involved in CK2β stabilization [23]. All variants of CK2βtes-like subunits preserve zinc fingers with cysteines (Fig. S3) that are responsible for dimer CK2β formation and its association with catalytic subunit [10]. CK2β is reminiscent of cyclins that are regulatory subunits of cyclin-dependent kinases and has a motif involved in regulation of cyclin degradation. Significant similarity is observed in degradation motif DKENTGLN [9] in different CK2βtes subunits, the KFNL sequence is preserved in CK2βtes subunits encoded by unique autosomal and amplified *CK2βtes-like* genes but not in Stellate. The acidic loop of CK2β is involved in regulation of catalytic subunit activity by modulating polyamine binding [9]. The DPEFDNED motif of acidic loop is significantly varied in CK2βtes proteins: the number of acidic residues in duplicated X-linked CK2βtes-like subunits is reduced to two residues compared to four residues in autosomal CK2βtes subunits encoded by unique genes. Possibly, these differences may be related to the peculiarities of functional modulations of the activity of these proteins.

The degree of nucleotide similarity between coding region of *CK2βtes*-*like* pairs GM17552/GM17570, GM17555/GM17552 and GD15860/GD24508 of paralogs approximates 83–86%. The extent of interspecific similarity between pair of orthologous copies GD15860/GM17570 and GD24508/GM17552 approximates 93% and 95%, respectively. Two paralogs, GM17552 and GM17556, in *D. sechellia* as well as the ortholog GD24508 in *D. simulans* are characterized by quite similar patterns of nucleotide substitutions (Fig. 1, Fig. S4). This similarity may be explained by duplication of the ancestor gene GM17552 and formation of a new copy GM17556 in *D. sechellia*. We found two practically identical *CK2βtes-like* copies in *D. sechellia* (GM17557a, GM17557b) separated by a sequence containing *DINE-1* fragments (Fig. 1, Fig. S4). We also detected a fragment of *CK2βtes*-*like* gene in *D. sechellia* and a vestige of its presence in *D. simulans* in a syntenic site where *Stellate* cluster has been formed in *D. melanogaster* (Fig. 1, Fig. S4).

### Origination of gene *Stellate*, a new variant of the *CK2βtes* gene family

**Figure 2:**
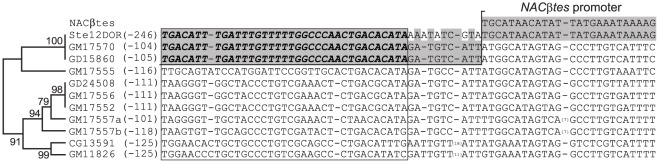
Recombination between the ancestor *CK2βtes*-*like* gene (GD15860 or GM17570) and *NACβtes* promoter region. Signature sequence of putative *CK2βtes*-like partner is designated in bold italics. The distances in nucleotides from the start of signature sequence and ORF start are indicated in brackets. Broken line shows the site of fusion of the *CK2βtes*-like and *NACβtes* sequences as a result of recombination. The tree represents the similarity of the nucleotide sequences in the selected box measured as the number of base differences [42] and was constructed using the UPGMA method [43]. The percentage of replicate trees in which the associated sequence clustered together in the bootstrap test (500 iterations) are shown next to the branches. Branches corresponding to partitions reproduced in less than 50% bootstrap replicates are collapsed.

The coding region of testis specific *Stellate* genes in *D. melanogaster* are homologous to the unique autosomal *CK2βtes* gene [14], [15], but *Stellate* precursor has acquired a promoter region from the *NACβtes* gene [20]. A careful comparison of nucleotide sequences of *Stellate* and *CK2βtes-like* genes in *D. sechellia* and *D. simulans* revealed the shared diagnostic sequence stretch between *Stellates* and orthologs GD15860/GM17570. This sequence is missed in all the other *CK2βtes-like* copies (Fig. 2). This observation allows us to consider the ancestor GD15860/GM17570-like copy to be a partner of illegitimate recombination with *NACßtes* gene (Fig. 2). The *CK2βtes*-*like* genes in *D. simulans/D. sechellia* (GD15860/GM17570) and *NACßtes* (CG13402) in *D. melanogaster* are located precisely at the same sites adjacent to orthologs GD17153, GM17653 and CG12480, respectively (Fig. 1). We suppose that the ancestor genome contained the juxtaposed *CK2βtes*-*like* and *NACβtes* genes at this site and such an arrangement allowed for recombination between these genes ensuring the emergence of the *Stellate* precursor copy.

The location of the *CK2ßtes-like* pseudogene in *D. sechellia* coincides with the site of the emergence of tandemly repeated *Stellate* cluster (Fig. 1). We propose that evolutionary diversification of genes related to *CK2ßtes* family has been occurred specifically in this specific region of the ancestor genome. These events appear to be quenched in *D. simulans/D. sechellia* lineage, but have led to the formation of *Stellate* cluster in *D. melanogaster*. The similarity of the tandemly repeated ORFs of novel young *Stellate* genes (2,5% divergency), which may be maintained by an unknown mechanism of homogenization [24], [25], is significantly higher than the extent of similarity of the homologous more ancient *CK2ßtes*-*like* copies in *D. sechellia/D. simulans* (Fig. S3, Fig. S4).

We detected an expansion of genes *CK2ßtes* and *NACßtes* by duplications. The usual fate of a gene duplicate is pseudogenization, but that has not occurred for most amplified *NACβtes* and *CKβ2tes-like* copies. Only one of six *NACßtes* copies in *D. melanogaster* is a pseudogene, located on the X-chromosome outside of this syntenic region, and only one *CK2βtes*-*like* pseudogene of six undamaged *CK2βtes*-*like* genes in *D.sechellia* is observed. Thus most duplicate copies remain functional.

**Figure 3:**
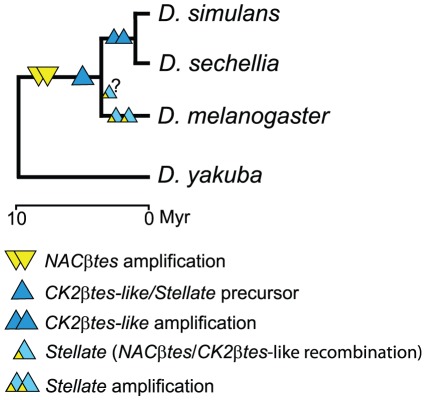
Fate of multigene families in the course of the divergence of *melanogaster* group species.

To summarize the obtained data, we present a chronology of the events of the *NACβtes* and *CK2βtes-like* genes amplification as well as *Stellate* origination related to the evolutionary tree of *melanogaste*r group species (Fig. 3). It is evident that amplification events of *NACβtes* genes and insertion of a precursor of *CK2βtes-lik/Stellate|* genes on the X-chromosome have been occurred in the common ancestor of *D. melanogaste*r, *D. simulans* and *D. sechellia*. The *CK2βtes-like* and *NACβtes* genes recombination that has led to the emergence of the *Stellate* genes is supposed to be proceeded in an immediate ancestor of *D. melanogaster*. Amplification of the *CK2βtes-like* genes has been originated in the common ancestor of *D. simulans* and *D. sechellia*.

### *DINE-1* transposons and expansion of the *CK2βtes* and *NACβtes* genes

Most genes from the *CK2βtes-like* and *NACβtes* families are flanked by *DINE-1* copies (Fig. 1). It has been reported that the evolution of new genes in *Drosophila* genomes is often associated with the abundant *DINE-1* transposons [6], [7], [26] related to the enigmatic *Helitron* family of transposable elements [27]–[34]. Our results support this view, providing examples of nonrandom *DINE-1*s localization near the amplified members of multigene families evolved in the course of evolution of the *melanogaster* subgroup genomes. The estimation of association of paralogs with *DINE-1* elements in *D. melanogaser* argues in favor of this view: 1180 genes grouped in 344 paralog families are known in *D. melanogaster*, and the fraction of paralogs having at least one *DINE-1* within 3 kb flanking sequences is significantly higher than can be expected by chance (243 vs. 156, *P-value*<0.005).

*DINE-1* transposons are thought to have invaded the *Drosophila* genome before the diversification of the *melanogaster* subgroup [27], [35]. It seems that *DINE-1* has gone through multiple independent cycles of activation and suppression [26]. These elements were suggested to be active and then silenced in the common ancestor of *melanogaster* subgroup species. *D. yakuba* is the only species showing evidence of a second, recent transpositional burst [35]. *D. melanogaster* and *D. sechellia/D. simulans* contain highly polymorphic *DINE-1* copies represented by the remnants of parent copies. The absence of nearly identical *Helitrons* at different loci in one genome indicates that these elements have been silenced for a long time and have undergone significant disruption processes [35]. Nevertheless, the analysis of the generalized structures of *DINE-1* sequences from 12 *Drosophila* genomes allowed the authors to discriminate some consensus regions including 5′- and 3′-subterminal inverted repeats, a core, and a 3′-terminal region containing a stem-loop structure that is supposed to be involved in the termination of *DINE-1* replication [26]. Using this consensus we were able to detect several profoundly damaged *DINE-1* copies in *D. melanogaster, D. sechellia and D. simulans*, adjacent to genes related to two studied multigene families (Fig. 1).

**Figure 4:**
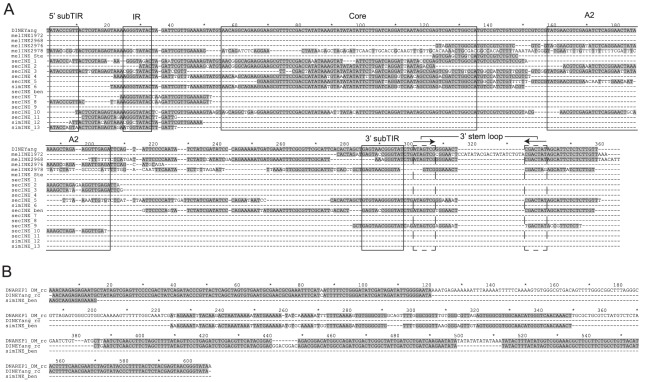
Multiple alignment of *DINE-1*copies in syntenic regions of *D. melanogaster* and *D. simulans/D. sechellia*. (**A**) Alignment of known and novel *DINE-1* copies with *D. melanogaster DINE-1* consensus sequence (DINEYang) [26]; consensus regions are designated according to [26]; (**B**) Alignment of the *simINE_ben* and *DNAREP1_DM* consensus sequence [36].

Alignment of nucleotide sequences of *DINE-1* copies and *D. melanogaster* consensus sequence [26] is shown in Fig. 4A. Although there are no extended shared regions between some copies (for example, between *INE2976* and *INE297*8), their relation to *DINE-1* is clearly traced by a comparison with the consensus sequence [26]. The relation of *simINE_ben* to *DINE-1s* is validated by its comparison to the earlier version of *DINE*-1 consensus [36] (Fig. 4B). The vestiges of *DINE-1s* flanking *NACβtes* duplications are detectable in both *D. sechellia* and *D. simulans* (Fig. 4A), confirming the presence of *DINE-1*s in the common ancestor of *D. melanogaster* and *D. sechellia/D. simulans*. The *CK2βtes*-*like* solo copies (GM17570 and GD15860) as well as the duplicated ones are located adjacent to damaged *DINE-1* sequences in *D. simulans/D. sechellia* (Fig. 1, Fig. 4, Table S1) at the distances not exceeding ∼200–1000 bp. Interestingly, the *ψNACβtes* (CR42877) located at a distance of ∼1 Mb from the studied region in *D. melanogaster* is also juxtaposed to a *DINE-1* copy.

Two non-homologous fragments of *DINE-1* flank the *Stellate* cluster (Fig. 1, Fig. 4A). The nucleotide sequence of the cluster including the distal marginal *Stellate* copy (CG33247), which is distinct in its 3′-noncoding region from the adjacent homogeneous tandem *Stellate* repeats, is identical to the “*Stellate* orphon” (*Ste12D OR*) located near the *ben* gene (Fig. 1). The observed identity of *Ste12D OR* and marginal *Stellate* copy (CG33247) in cluster (Fig. S3) allows us to propose the role of *DINE-1*s in duplication of *Ste12D OR* followed by its local amplification to generate the *Stellate* cluster. While the sequences of the orphon and marginal *Stellate* copies are identical to each other, the adjacent *DINE-1* copies (*INE*1972 and *INE*2968) contain similar 3′-stem-loop sequences, but have been deeply disrupted in the rest of the *DINE-1* sequence. We propose that diverged *DINE-1* copies may participate in the ancestor genomes causing non-allelic recombination that is capable to ensure reshuffling of protein coding genes. Alternatively, *DINE-1* sequences may be prone to breakages followed by illegitimate recombination [6]. Thus *DINE-1* participation in evolution of multigene families remains to be mysterious.

While the precise testis specific functions of the members of both multigene families remain unknown, positive selection has been shown for *NACßtes* genes [37]. At the same time, the involvement of *DINE-1* in duplication of the testis specific *kep1* gene followed by formation of a young gene implicated in regulation of the Y-linked male fertility genes has been demonstrated [7]. The elucidation of *CK2ßtes* and *NACßtes* gene functions in testes will help to understand whether there is an evolutionary benefit to their expansion and coupled evolution in *Drosophila* species.

## Materials and Methods

The gene annotation of *D. melanogaster* (r5.35), *D. sechellia* (r1.3), *D. simulans* (r1.3) and *D. yakuba* (r1.3) is according to FlyBase (http://flybase.org/). The degree of nucleotide similarity between coding regions of *CK2βtes* family genes was evaluated by BLAST (v. 2.2.26) [38]. All alignments were performed by ClustalW implemented in Vector NTI program (Invitrogen).

The identification of novel *DINE-1*s in the *D. simulans/D. sechellia* genomes was performed by BLAST (v. 2.2.21) [38] using the *DINE-1* consensus sequences [26], [36] as queries. The found candidate fragments of *DINE-1s* copies were additionally reverse BLASTed against *D. melanogaster* genome assembly to check if they are matched to known *INE-1* repeats only. The evolutionary history of proteins related to CK2βtes family was inferred by using the Maximum Likelihood method based on the JTT matrix-based model [39]. All positions containing gaps and missing data were eliminated. The resulted tree is a bootstrap consensus tree inferred from 500 replicates [40]. Evolutionary analyses were conducted in MEGA5 [41].

The list of *D. melanogaster* paralogs was fetched from HomoloGene NCBI database (http://www.ncbi.nlm.nih.gov/homologene). The expected number of paralogs with nearby *DINE-1*s was calculated as a possibility to find the *DINE-1* near the gene (total number of *DINE-1*s located within 3 kb of RefSeq gene flanks divided to the total number of all RefSeq genes) magnified to the total number of paralogs. Statistical significance of difference between the expected and observed numbers of paralogs were checked by Chi-square test. The genes and *DINE-1s* on chromosomes U and Uextra were not taken into account.

RT-PCR was carried out using RNA from testes, heads and carcasses of adult flies of *D. simulans* (stock 199 from Bloomington Stock Center). Total RNA was extracted by Trizol reagent (Invitrogene), and first strand cDNA synthesis was performed by using oligo(dT) primer and SuperScript II reverse transcriptase (Invitrogen). Sequences of the used primers are 5′-GCTGTAACGACGTCTTCAAGC-3′ (GD24508_F) and 5′-ATTCGCAATCGAGGACTCGC-3′ (GD24508_R). The PCR products were sequenced for verification of their specificity.

## Supporting Information

Figure S1 **Pair alignment of the** ***NACβtes*** **gene and pseudogene sequences of** ***D. sechellia*** **.** ψ*NACßtes* is localized in *D. sechellia* scaffold_20:807538..808222[-].
(EPS)

Figure S2 **RT-PCR validation of testis expression of** ***CK2βtes*** **-** ***like*** **GD24508 gene in** ***D. simulans*** **.** Lanes: 1, 100 bp marker; 2, total DNA; 3, 4 and 5, RNA from testes, heads, and carcasses of adult males, respectively. Specificity of PCR products was confirmed by sequencing. Designated primers flank second small intron (∼50 nt).
(EPS)

Figure S3 **Analysis of proteins related to CK2βtes family.** (**A**) Multiple alignment of CK2βtes proteins. Black spots depict serine phosphorylation sites, asterisks depict zinc-finger cysteine residues. GE11447, GM11826 and CG13591 are autosomal unique *CK2βtes* genes in *D. yakuba*, *D. sechellia* and *D. melanogaster*, respectively. (**B**) Molecular phylogenetic analysis of CK2βtes proteins inferred by Maximum Likelihood method. The percentage of replicate trees in which the associated proteins clustered together in the bootstrap test is shown near the branches. Initial tree for the heuristic search were obtained automatically as follows: when the number of common sites was <100 or less than one fourth of the total number of sites, the maximum parsimony method was used; otherwise BIONJ method with MCL distance matrix was used. The tree is drawn to scale, with branch lengths measured in the number of substitutions per site. There were a total of 154 positions of the 11 amino acid sequences in the final dataset.
(EPS)

Figure S4 **Multiple alignment for nucleotide sequences encompassing exon1, intron and a fragment of exon 2 of** ***CK2ßtes*** **genes.** The designations of genes are the same as in Fig. 1.
(EPS)

Table S1 **Location of** ***DINE-1s*** **and nearby genes.**
(PDF)
