# Supplementary material for: Expansion and Evolution of the X-Linked Testis Specific Multigene Families in the melanogaster Species Subgroup
Source: PLoS One. 2012 May 23;7(5):e37738. doi: 10.1371/journal.pone.0037738 (PMC3359341; doi:10.1371/journal.pone.0037738)
Supplement: Table S1 — Location of DINE-1s and nearby genes. (PDF) [file pone.0037738.s005.pdf]

Table S1.

DINE name	DINE location	Nearest gene	Nearest gene location
secINE_1	scaffold_20:804385..804525 (-)	GM17556	scaffold_20:805648..804852 (-)
secINE_2	scaffold_20:805871..806008 (+)	GM17556	scaffold_20:805648..804852 (-)
secINE_3	scaffold_20:807384..807556 (+)	GM17555	scaffold_20:806953..806301 (-)
secINE_4	scaffold_20:810523..810652 (+)	GM17553	scaffold_20:809591..808941 (-)
secINE_5	scaffold_20:811995..812234 (+)	GM17552	scaffold_20:811749..810953 (-)
simINE_6	chrX_Mrandom_708:7610..7739 (+)	GD24509	chrX_Mrandom_708:6761..6003 (-)
secINE_7	scaffold_20:786211..786239 (+)	GM17557	scaffold_20:786724..790932 (-)
secINE_8	scaffold_20:789164..789206 (+)	GM17557	scaffold_20:786724..790932 (-)
secINE_9	scaffold_20:789302..789352 (+)	-	
secINE_10	scaffold_20:789649..789832 (+)	GM17557	scaffold_20:786724..790932 (-)
secINE_11	scaffold_20:670270..670298 (+)	GM17570	Scaffold_20:670653..671360 (-)
simINE_12	chrX:10811480..10811520 (-)	GD15860	chrX:10811247..10810547 (-)
simINE_13	chrX:10809903..10809941 (+)	GD15860	chrX:10811247..10810547 (-)
melINE2968	chrX:13940441..13940567 (+)	CG33247	chrX:13941528..13940805 (-)
melINE1972	chrX:13895066..13895151 (-)	Ste12DOR	chrX:13894243..13894924 (+)
melINE2976	chrX:14208354..14208441 (+)	CG18157	chrX:14209616..14208656 (-)
melINE2978	chrX:14211480..14211861 (+)	CG32598	chrX:14211183..14210159 (-)
melINE_Ste1	chrX:13955559..13955586 (+)	CG33236	chrX:13955458..13954735 (-)
simINE_ben	chrX:10697861..10697979 (+)	GD17149	chrX:10696559..10696104 (+)
secINE_ben	scaffold_20:534842..534954 (-)	GD17649	scaffold_20:533142..533594 (+)
